# Integrated multiomic approach for identification of novel immunotherapeutic targets in AML

**DOI:** 10.1186/s40364-022-00390-4

**Published:** 2022-06-10

**Authors:** Thomas Köhnke, Xilong Liu, Sascha Haubner, Veit Bücklein, Gerulf Hänel, Christina Krupka, Victor Solis-Mezarino, Franz Herzog, Marion Subklewe

**Affiliations:** 1 Department of Medicine III, University Hospital, LMU Munich, Marchioninistr 15, 81377 Munich, Germany; 2 Laboratory for Translational Cancer Immunology, Gene Center Munich, LMU Munich, Munich, Germany; 3 Department of Biochemistry, Gene Center, LMU Munich, Munich, Germany; 4 German Cancer Consortium (DKTK), Heidelberg, Germany; 5 German Cancer Research Center (DKFZ), Heidelberg, Germany

**Keywords:** Acute myeloid leukemia, Immunology, Proteomics, Leukemia

## Abstract

**Background:**

Immunotherapy of acute myeloid leukemia has experienced considerable advances, however novel target antigens continue to be sought after. To this end, unbiased approaches for surface protein detection are limited and integration with other data types, such as gene expression and somatic mutational burden, are poorly utilized. The Cell Surface Capture technology provides an unbiased, discovery-driven approach to map the surface proteins on cells of interest. Yet, direct utilization of primary patient samples has been limited by the considerable number of viable cells needed.

**Methods:**

Here, we optimized the Cell Surface Capture protocol to enable direct interrogation of primary patient samples and applied our optimized protocol to a set of samples from patients with acute myeloid leukemia (AML) to generate the AML surfaceome. We then further curated this AML surfaceome to exclude antigens expressed on healthy tissues and integrated mutational burden data from hematologic cancers to further enrich for targets which are likely to be essential to leukemia biology. Finally, we validated our findings in a separate cohort of AML patient samples.

**Results:**

Our protocol modifications allowed us to double the yield in identified proteins and increased the specificity from 54 to 80.4% compared to previous approaches. Using primary AML patient samples, we were able to identify a total of 621 surface proteins comprising the AML surfaceome. We integrated this data with gene expression and mutational burden data to curate a set of robust putative target antigens. Seventy-six proteins were selected as potential candidates for further investigation of which we validated the most promising novel candidate markers, and identified CD148, ITGA4 and Integrin beta-7 as promising targets in AML. Integrin beta-7 showed the most promising combination of expression in patient AML samples, and low or absent expression on healthy hematopoietic tissue.

**Conclusion:**

Taken together, we demonstrate the feasibility of a highly optimized surfaceome detection method to interrogate the entire AML surfaceome directly from primary patient samples and integrate this data with gene expression and mutational burden data to achieve a robust, multiomic target identification platform. This approach has the potential to accelerate the unbiased target identification for immunotherapy of AML.

**Supplementary Information:**

The online version contains supplementary material available at 10.1186/s40364-022-00390-4.

## Background

Immunotherapeutic strategies including chimeric antigen receptor (CAR) T cells or antibody based therapies have transformed treatment outcomes in cancer. For acute myeloid leukemia (AML) however, while there have been considerable advances in the field of immunotherapy, lack of efficacy or on-target off-leukemia toxicity remain a challenge. Several studies have recently demonstrated patterns of immune-activation and -evasion in AML [1, 2], highlighting the complex interaction of genomic lesions and immune activation. However, despite these advances, identification of suitable targets have remained challenging. Thus, novel target antigens with more favorable expression characteristics are sought after. Genome-wide assessment of mutational burden and gene expression directly from primary patient samples have delivered significant insight into AML biology and prognosis. However, only recent technical advances in proteomics have allowed for unbiased and sensitive analysis of the cancer proteome. For the development of novel immunotherapeutic strategies, the choice of which surface antigen to target is arguably the most important design step. Efficient and unbiased interrogation of the entirety of surface proteins has been technically challenging. While previous reports have characterized the AML surfaceome in cell lines [3, 4], cell-surface capture proteomics directly on primary patient samples has only been successfully performed in xeno-amplified acute lymphoblastic leukemia [5]. However, both clonal composition as well as surface antigen expression have been reported to change upon xenotransplantation in AML [6, 7]. Other approaches aiming to increase throughput of surface protein detection have either adapted traditional flow cytometry [8] or mass cytometry based approaches [9]. However, these methodologies are dependent on the existence of antibodies against a-priori defined target antigens, outlining inherent limitations for de novo target discovery. We therefore aimed to close this gap by improving the current methodologies to enable unbiased, whole-surfaceome detection directly from xeno-amplification free AML patient samples.

Additionally, while several studies have incorporated gene expression data to identify putative immunotherapeutic targets [3], gene sequencing of somatic mutations contributed significantly to our understanding of underlying biologic mechanisms of leukemogenesis [10]. These sequencing efforts have been performed in large patient cohorts, primarily with the goal to identify drivers of disease. However, this data type has not been systematically incorporated into the discovery of immunotherapeutic targets. We believe integration into target-discovery platforms should improve their robustness, since effective immunotherapeutic targets ought not to only be expressed uniformly, these targets should also have *functional* relevance to the tumor, thus *rarely* be deleted or affected by change-of-function variants. We postulate that mutation data might be incorporated into a target discovery platform and implement a strategy to achieve this goal.

In this study, we optimized a targeted proteomics assay for the efficient interrogation of the entire surface proteome (surfaceome) of primary acute myeloid leukemia samples without the necessity of xenograft amplification. To avoid xenotransplantation, we employed our short-term *in vitro* culturing system, which we previously demonstrated does not show large shifts in clonal composition or antigen expression [11]. Next, we integrated our proteomics data into a comprehensive set of gene expression as well as mutational burden data to enrich for expressed and functionally relevant targets. We then validated a set of high-scoring putative targets identified by our approach in independent primary AML samples.

This integrated approach allows for unbiased, genome-wide screening from all three modalities – mutation, transcription and surface protein expression – for target discovery in immunotherapy of AML.

## Methods

### Cell culture

HL-60 and OCI-AML3 AML cell lines were grown in RPMI 1640 medium plus 10% fetal bovine serum (FBS), 1% glutamine, 1% penicillin/streptomycin and 5 µM β-mercaptoethanol at 37 °C and 5% CO_2_.

### Primary patient cell culture

Peripheral blood (PB) or bone marrow (BM) samples were collected at the Laboratory of Leukemia Diagnostics at the University Hospital Munich with Institutional Review Board approval. Ficoll density gradient centrifugation was performed to isolate mononuclear cells and patient specimens were subsequently stored in liquid nitrogen until further use. *In vitro* culture of patient samples was performed as previously reported [12]. Specifically, irradiated MS-5 stromal cells were seeded in 6-well plates at 2.5 × 10^5^ cells/well 1 day prior to addition of patient specimens in α-MEM with 12.5% FBS, 12.5% horse serum, 1% glutamine, and 1% penicillin/streptomycin. 1–1.5 × 10^7^ primary patient cells per well were added into the plate and the media was supplemented with IL-3, TPO and G-CSF (all 20 ng/ml). After 3 days, the non-adherent cells were subjected to the Glyco-CSC protocol as described below. Detailed patient information is summarized in Table S1.

### Patient-derived xenograft (PDX) cells

Patient-derived xenograft (PDX) cells were collected from mice as previously described by Vick et al. [13]. Specifically, primary AML patient mononuclear cells were transplanted i.v. into sublethally irradiated NSG mice. After 6–20 weeks, prior to mice showing clinical signs of illness, mice were euthanized and human cells were isolated from spleen and bone marrow.

### Cell Surface Capture (CSC)

The original Glyco-CSC, Cys-Glyco-CSC as well as Lys-CSC protocols were performed as described by Wollscheid et al. as well as Bausch-Fluck et al. [14, 15]. Specifically, 1 × 10^8^ cells were selectively labeled after sodium-meta-periodate oxidation with biocytin hydrazide (for Glyco-CSC and Cys-Glyco-CSC protocols, Biomol) or directly without oxidation using NHS-SS-biotin (Lys-CSC protocol, ThermoFisher). Then, cells were lysed in hypotonic lysis buffer with a Dounce homogenizer on ice. Cell debris was removed by centrifugation and the supernatant (containing the membrane fraction) collected. Next, the cell membrane fraction was subjected to ultracentrifugation either in a buffer consisting of a 1:1 ratio with lysis buffer and membrane preparation buffer (for Glyco-CSC and Cys-Glyco-CSC), or in a sucrose gradient (Lys-CSC). The resulting membrane pellets were washed and dissolved in ammonium bicarbonate solution by indirect sonication.

For the Glyco-CSC protocol, the membrane proteins were reduced using tris(2-carboxyethyl)phosphine and then alkylated by iodoacetamide. Membrane proteins were digested overnight with trypsin (Promega). The biotinylated membrane proteins were then bound to a Streptavidin Plus UltraLink Resin, washed and either released by PNGase F (Glyco-CSC and Cys-Glyco-CSC) or by reducing agents (cysteine contained peptides in Cys-Glyco-CSC and Lys-CSC). Peptides were desalted and washed in C18 columns (ThermoFisher) and concentrated in a SpeedVac concentrator (Uniequip). Lastly, dried peptides were resuspended in 20 µl LC–MS grade water (supplemented with 0.1% formic acid and 5% acetonitrile) and stored at -20 °C.

### Modifications to the CSC protocol

To improve the CSC protocol and enable direct utilization of primary patients specimens, we modified the original protocols to incorporate the following changes: the concentration of biocytin hydrazide was reduced to 5.4 mM (original 6.5 mM); samples were homogenized using a Bioruptor device (Diagenode) instead of a dounce homogenizer; trypsin digestion was performed twice; and finally the NH_4_HCO_3_ solution was replaced with a digestion buffer (containing 1 mM iodoacetamide, 100 mM NH_4_HCO_3_, and 1 mM BHES for Glyco-CSC and Cys-Glyco-CSC, or 1.25 mM iodoacetamide, 100 mM NH4HCO3, 2 mg/ml RapiGest surfactant, and 1.25 mM BHES for Lys-CSC).

### Liquid chromatography–mass spectrometry

Peptide samples were separated by a 70 min gradient on a reversed-phase nano HPLC and analyzed by an Orbitrap Elite instrument. MS1 spectra were acquired at 120,000 resolution in data-dependent acquisition mode (top 10) and MS2 spectra at low resolution in the ion trap as well as the following settings: activation—10 ms; maximum injection time—100 ms; AGC target value—104 ions; and minimum ion count—500. Each sample was analyzed in duplicate and technical replicates were averaged.

Raw data files were loaded into MaxQuant for protein identification against the reviewed human proteome (UniProt August-2015). Maximum precursor mass error was set to 4.5 ppm and the fragment mass error was 0.5 Da. The following search parameters were used as variable modification: protein N-terminal acetylation, methionine oxidation and N-deamidation. Carbamidomethylation on cysteines was set as a fixed modification. The final peptide and protein lists were filtered using a false discovery rate (FDR) of 0.01.

Next, the results were further refined using annotations from the Cell Surface Protein Atlas and UniProt. Specifically, we validated deamidation events as N-glycosylation if they fulfilled the following criteria: 1) the deamidation had a MaxQuant localization probability > 0.75 and the deamidation occurred in a NXS/T glycosylation motif; 2) the identified protein contained at least one transmembrane (TM) domain and/or signal peptide; and 3) the NXS/T glycosylation motif did not occur within the TM domain. Intensities between different samples were compared at the deamidation site level as well as protein level and intensities of N-glycosylations of the same site but in different tryptic peptides were summed. N-glycosylations at the same protein were averaged. Finally, intensities were log2-transformed and normalized to the median between samples. Protein level clustering was performed by hierarchical clustering either using Cosine correlation distance or Euclidean distance. Edges with a cosine correlation lower than 0.85 in the network plot were filtered out. Each sample was tested at least twice and intensities of the identified peptides were averaged.

### Filtering of putative targets

Next, we filtered our list of putative surfaceome targets further to eliminate genes that fulfilled the were either highly expressed on healthy hematopoietic cells and/or highly expressed on other normal human tissues. Specifically, we utilized two gene expression databases: one derived from healthy blood cells in different stages of hematopoiesis (BloodSpot—HemaExplorer) and one derived from normal human tissues (Genotype-Tissue Expression Project (GTEx)). The mean and standard deviation for all of our surfaceome genes was enumerated and those genes with expression on three hematopoietic subpopulations (hematopoietic stem cells, early hematopoietic progenitors and CD14 + monocytes) beyond the upper standard deviation of the mean were eliminated (Supplementary Figure S1A). Similarly, the maximal tissue expression on non-hematologic tissue was enumerated for our surfaceome genes and those genes, where the maximal expression found in any non-hematologic tissue was beyond the upper standard deviation of the mean were eliminated (Supplementary Figure S1B).

### Mutational burden analysis

Somatic mutations were downloaded from the Catalogue Of Somatic Mutations In Cancer (COSMIC, V91). Tumor types were grouped in non-hematologic malignancies (Non-Heme) and hematologic malignancies (Heme). Finally, the ratio of non-synonymous vs. synonymous mutations (dN/dS-ratio) was enumerated for all genes detected in our AML surfaceome.

### FACS analysis

Flow cytometric validation of our putative surfaceome target antigens was performed using an antibody panel shown in Supplementary Table S4. Independent AML patient samples were analyzed in the Laboratory of Leukemia Diagnostics at the University Hospital Munich. After thawing, patient samples were washed with PBS and 1 × 10^6^ cells were resuspended in 100 µl of FACS buffer. After staining with the appropriate amount of antibody or isotype control, cells were washed and data was acquired using a Navios flow cytometer (Beckman Coulter). Subpopulations were gated as outlined in Supplementary Figure S2. The target antigen expression levels were determined by median fluorescence intensity (MFI) ratio by dividing the MFI value of the antigen-specific antibody by the MFI value of the respective isotype control.

### Statistics

Venn Diagrams were generated using nVenn [16] and statistical analyses for significant differences between samples/groups were performed using unpaired two-tailed t-tests using GraphPad Prism 6, unless otherwise stated.

## Results

### Cell Surface Capture Technology

**Fig. 1:**
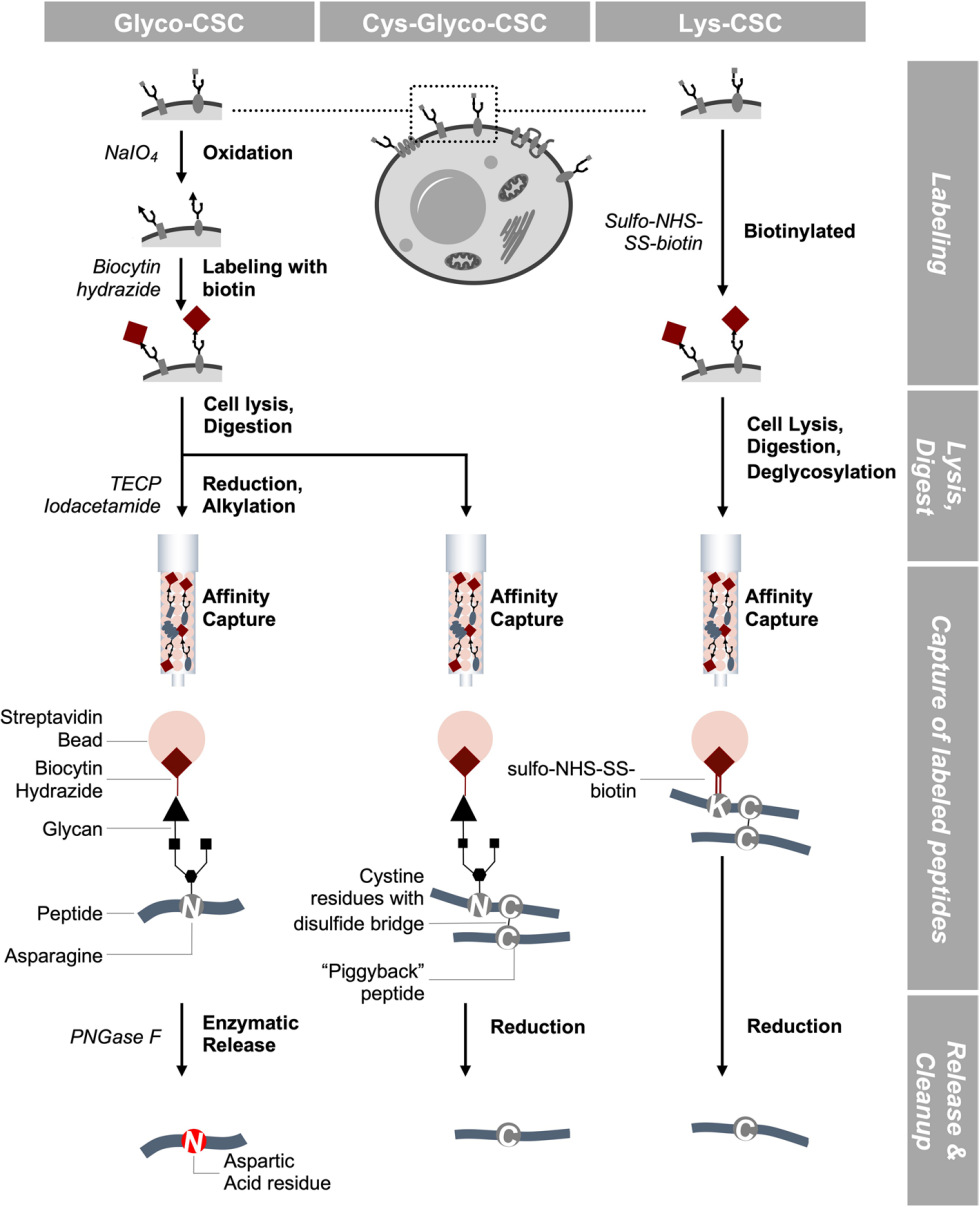
Outline of Glyco-Cell Surface Capture (CSC) and its variants Cys-Glyco-CSC and Lys-CSC. The workflows adapted from the protocols by Wollscheid et al. as well as Bausch-Fluck et al. [14, 15] and are comprised of labeling, digestion, affinity capture, peptide release and peptide identification by tandem mass spectrometry

In this study, we aimed to interrogate the AML surfaceome from primary AML samples. The technology, initially described by Wollscheid et al. [14], relies on the labelling of accessible glycosylation sites or Lysine residues (Fig. 1). In theory, this approach thus allows to characterize the entirety of expressed surface proteins without the need of antibodies or any knowledge of the expected targets. However, the most relevant limitation for the applicability in primary patient samples is that the established protocol requires substantial cell numbers in excess of 10^8^—10^9^ cells. We thus set out to optimize the protocol for the interrogation of AML cells.

### Protocol Optimization increases protein recovery

**Fig. 2:**
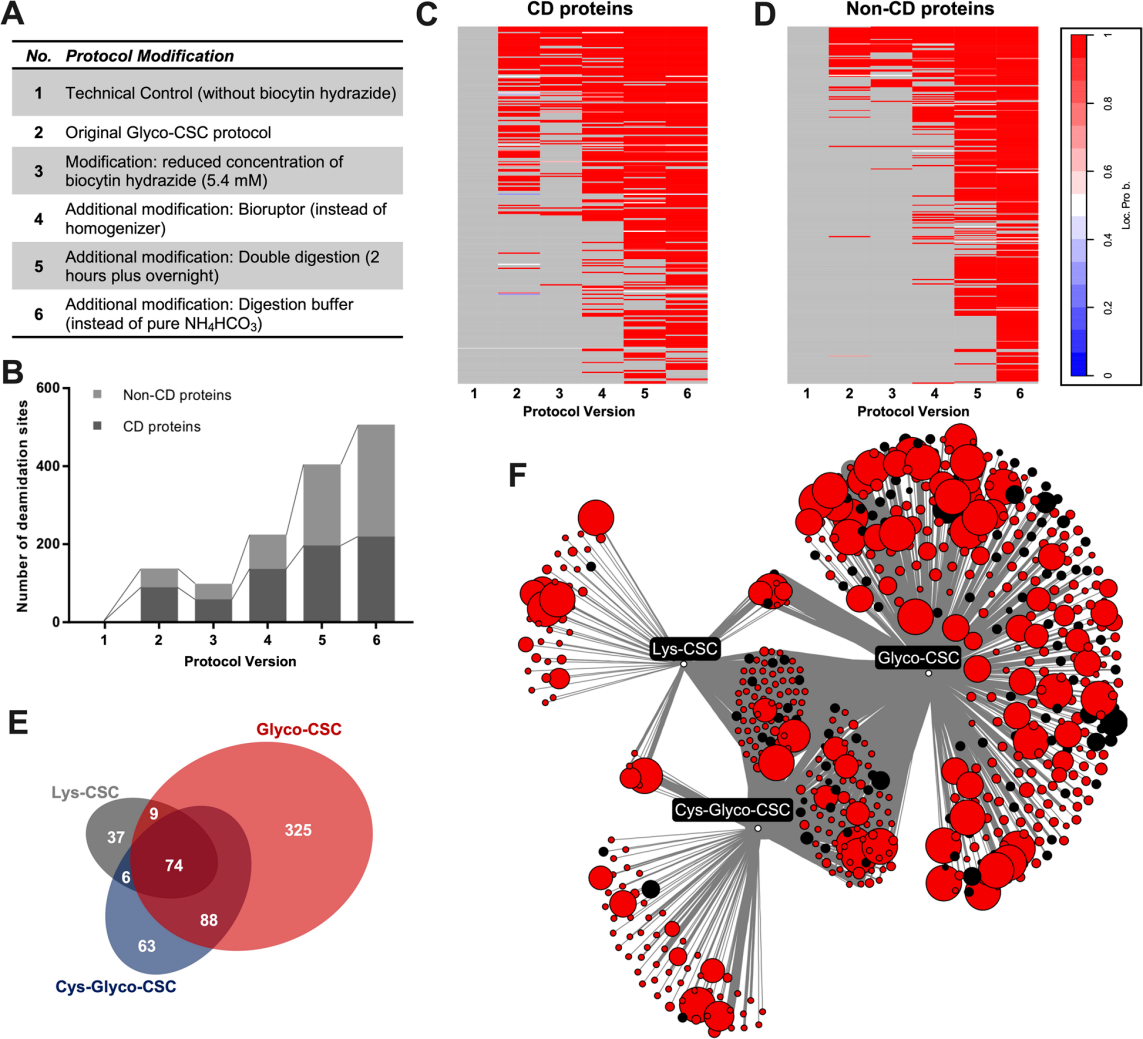
Protocol Optimization Steps using OCI-AML3 cells. **A** Protocol optimization steps for Glyco-CSC examined. **B** Bar graph of number of deamidation sites from the OCI-AML3 cell line according to our improved protocol versions. No. 6 yielded > 500 deamidation sites, representing 252 proteins. **C** & **D** Probability of N-glycosylation events being localized at the expected position of identified peptides in each group (Loc. Prob., threshold ≥ 0.75) for CD proteins (C) and Non-CD proteins (D). **E** Comparison of detected proteins between Glyco-CSC, Lys-CSC and Cys-Glyco-CSC. Numbers refer to detected proteins. **F** Detailed view of detected proteins in comparison experiment summarized in (E). Nodes representing CD proteins (black) and non-CD proteins (red). Thickness of connecting lines is proportional to the number of peptides identified for the associated protein and size of the node indicates the number of transmembrane domains (TMs). Proteins with ≥ 10 TMs were limited to 10

We set out to optimize the protocol by step-wise introduction of protocol modifications as outlined in Fig. 2A. While a slight reduction of the biocytin hydrazide concentration did not increase yield alone, together with improved mechanical homogenization the number of detected peptides increased by approx. 30% (Fig. 2B). However, the most pronounced improvement was detectable by including a second digestion step, and finally a modified digestion buffer markedly increased yield for both cluster of differentiation (CD) proteins (Fig. 2C) as well as Non-CD proteins (Fig. 2D). This highlights the importance of optimized digestion conditions for a maximum of mass spectrometric identifications. Importantly, these modifications to the original Glyco-CSC protocol also improved the specificity of the assay: We reasoned that peptides were likely specifically captured in the assay if they displayed the characteristic mass shift (0.984 Da) associated with successful N-glycosylation and the corresponding protein contained a transmembrane domain or signal peptide. Using this metric of specificity, 54% of all identified peptides in the unmodified Glyco-CSC protocol fulfilled these criteria. Notably, our modified protocol was able to increase this metric to 80.4%.

Next, we aimed to interrogate whether Glyco-CSC is sufficient to detect the majority of cell surface proteins or whether the CSC-variants Lys-CSC or Cys-Glyco-CSC drastically increase the number of identified proteins in AML. Using our modified protocol, we noted that the vast majority of proteins were identified using the Glyco-CSC technology, whereas the number of additional proteins identified by either Lys-CSC or Cys-Glyco-CSC were only modest (Fig. 2E and F). These two observations allowed us to conclude that our protocol modifications drastically increased the number of identified proteins and that the vast majority of proteins was obtained by the Glyco-CSC method.

### Our Modified CSC-workflow allows for the efficient capture of the AML surfaceome from primary patient samples

Next, we applied our modified Glyco-CSC methodology to primary patient samples. In previous studies in acute lymphoblastic leukemia, investigators (using the traditional Glyco-CSC protocol) used a xenoamplification strategy to obtain sufficient cell material for CSC [5]. We thus initially applied a similar strategy and obtained cells from two patient-derived AML xenograft samples and subjected them to our modified Glyco-CSC protocol. In parallel, our laboratory has established a robust culture system that allows for culturing and functional interrogation of primary AML samples *in vitro* [12]. We previously validated that this culture system retains antigen expression levels, preserves the relative frequency of genetic (sub)clones and retains the frequency of leukemia-initiating cells [11]. Given our extensive experience with this technique and our successful modifications of the Glyco-CSC protocol, we next subjected cells cultured *in vitro* for 3 days to the Glyco-CSC protocol.

**Fig. 3:**
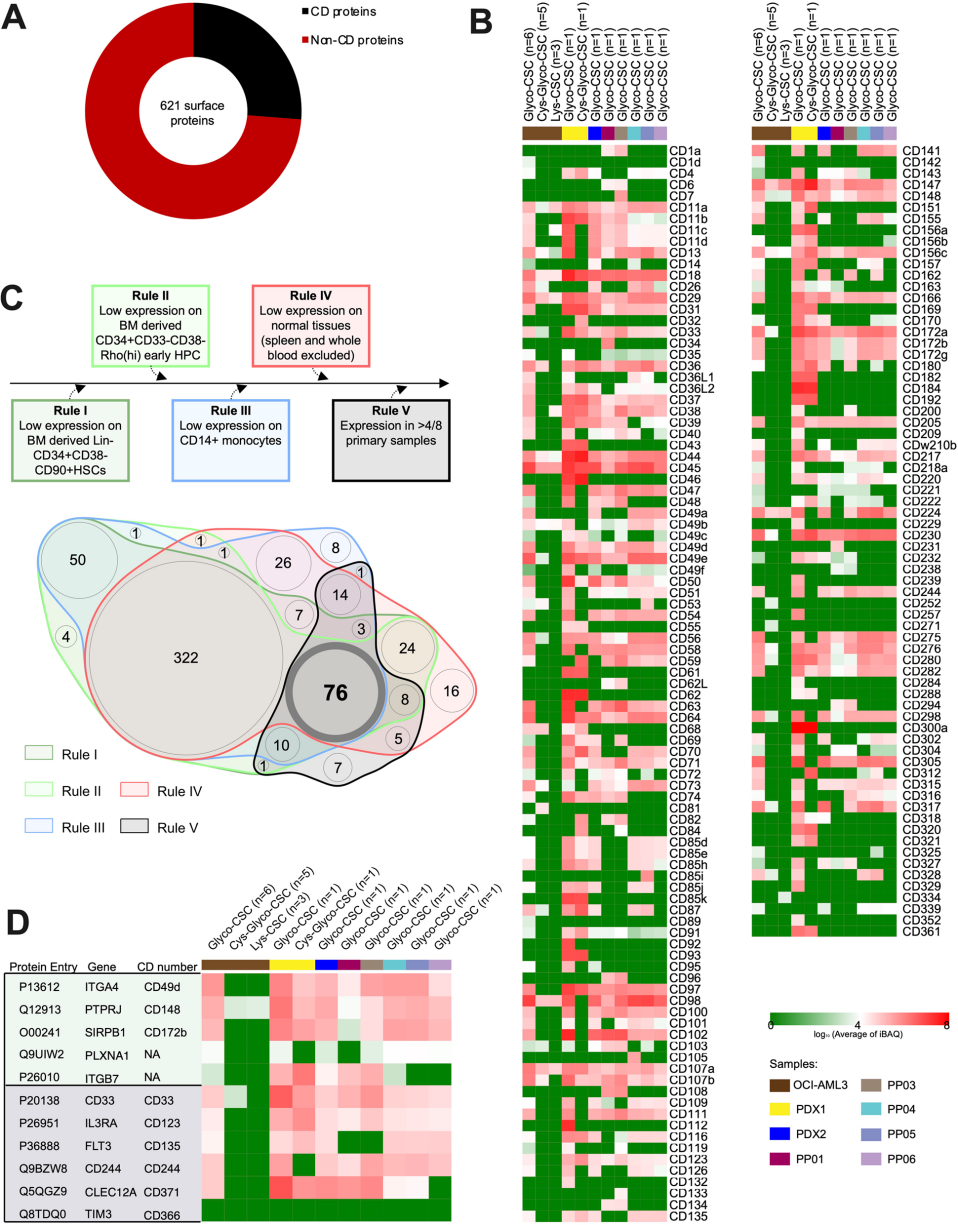
Modified CSC-workflow allows for the interrogation of the AML surfaceome in primary patient samples. **A** 621 surface proteins identified with our modified CSC technology 7 clinical samples and 1 AML cell line (OCI-AML3), separated into CD proteins (black, 163) and non-CD proteins (red, 458). **B** Expression levels of CD proteins identified by CSC. Heatmap intensity indicates the log10 average of iBAQ. **C** Overview of filtering strategy to eliminate targets with abundant expression on normal healthy tissue using publicly available gene expression databases. Furthermore, only proteins that were detected in at least half of the primary patient samples were considered. As a result, 76 proteins remain as potential candidates for manual evaluation. **D** Protein expression of 5 new putative targets (top, in green) and 6 markers currently being investigated (bottom, in grey) as immunotherapeutic targets in AML samples

Results for these experiments are summarized in Figs. 3A & B. In total, we identified 621 surface proteins on our primary patient samples. Importantly however, not all proteins were present on all samples (Fig. 3B). While the cell line experiments for OCI-AML3 cells (included in the heatmaps for reference) showed some overlap with the primary patient samples, we also identified surface proteins in several of the patient samples that were not present in the cell line data. Importantly, while the cell number obtained from the patient-derived xenografts was higher than what was available from short-term cultured primary patient material directly, our data shows detection of similar total numbers of proteins (Fig. 3B), highlighting that the combination of protocol improvements and our *in vitro* culturing conditions allows for the robust interrogation of the AML surfaceome directly from primary patient material. Of note, we observed a > 90% overlap in detected proteins in technical replicates, underlining the robustness of our technique (Supplementary Figure S3).

### Filtering of putative targets

Next we set out to identify a shortlist of potential targets for immunotherapy in AML. Unlike the B cell target antigen CD19, where significant on-target, off-tumor activity is well tolerated, targeting of antigens that are widely expressed on myeloid lineage cells is more problematic. The main concern in this context remains toxicity against healthy cells but also widely expressed antigens acting as an ‘antigen-sink’, which depletes the amount of bioavailable therapeutic agent [17]. We thus set out to eliminate antigens that are highly expressed on cells within the hematopoietic stem cell compartment, on mature monocytes and on non-hematopoietic tissues. Our approach involved the mining of publicly available gene expression data of these tissue types. We defined a set of 5 rules, which focused on different cellular compartments, where expression of our putative targets might limit therapeutic efficacy. These were immature hematopoietic stem cells as well as early hematopoietic progenitor cells, CD14 + monocytes, and non-hematopoietic normal tissues. Specifically, we determined expression levels of our surfaceome candidate genes in these hematologic subpopulations and eliminated those genes with expression levels beyond the upper standard deviation of the mean from our cohort (Supplementary Figure S1A). Finally, we required that the putative target needed to be detected in our Glyco-CSC surfaceome dataset in more than half of the primary patient samples investigated (Fig. 3C, top panel). Interestingly, the largest number of excluded targets was eliminated during this last step (322 targets), further illustrating that AML remains an immunophenotypically heterogeneous disease. Applying this set of filters to our dataset, we obtained a shortlist of 76 putative targets (Fig. 3C, bottom panel), which can now be subjected to manual curation and further validation (Supplementary Table 2). Of note, in our dataset, many immunotherapeutic targets currently investigated for the therapy of AML were also detected, including CD33, CD123 (IL3RA), and CLL-1 (CLEC12A), amongst others. Detection of our putative list of targets was robust across the set of samples investigated, with few outliers (Fig. 3D). The data for the AML Surfaceome and the respective gene expression values from healthy tissues are summarized in Supplementary Table 3.

### Mutational burden analysis

**Fig. 4:**
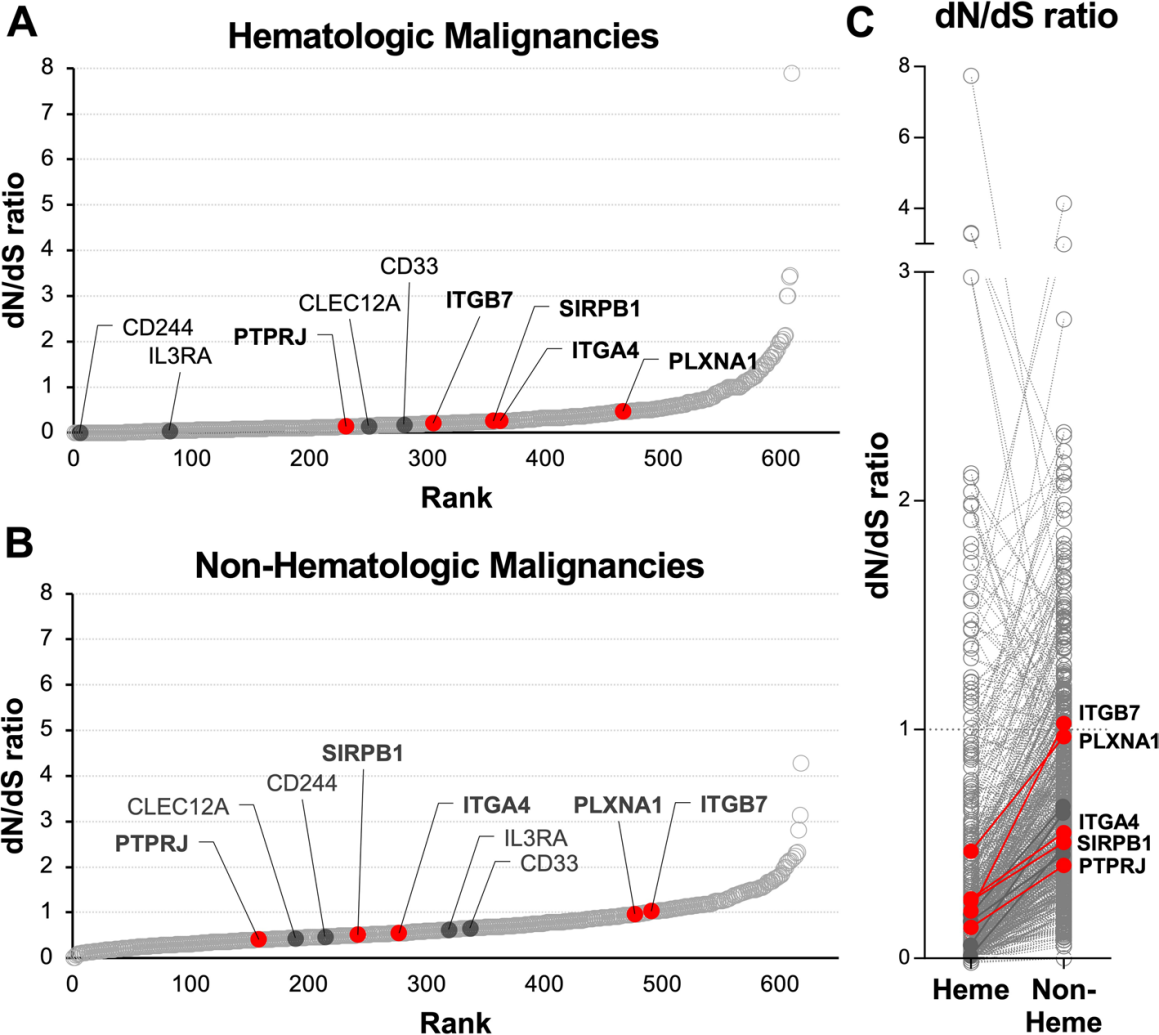
Candidate antigens display low frequency of non-synonymous mutations, suggesting their functional relevance in hematologic malignancies. **A** Ratio of Non-synonymous to synonymous mutations in hematologic malignancies found in genome-wide screens in the COSMIC database in genes represented in our surfaceome dataset. Genes are sorted by rank of dN/dS ratio. Selected immunotherapy targets are highlighted. **B** Ratio of Non-synonymous to synonymous mutations in Non-hematologic malignancies in the COSMIC database. Genes are the same as in A. **C** Pairwise comparison of relative Non-synonymous mutation rates amongst surfaceome genes between Hematologic and Non-Hematologic malignancies

To further scrutinize our list of detected surface proteins, we aimed to interrogate whether these genes are likely functionally relevant and thus expected to be of low risk to escape under therapeutic pressure. By enumerating the rate of nonsynonymous mutations observed in large patient cohorts, we aim to identify whether *expressed* genes of interest are *hypo*mutated and thus might serve an *essential* role in leukemic cell survival. We interrogated the database curated by the Catalogue Of Somatic Mutations In Cancer (COSMIC, V91) and specifically enumerated the non-synonymous vs. synonymous mutations (dN/dS-ratio) found in the set of genes identified in the AML surfaceome. We excluded the known driver genes in FLT3 and CALR for this analysis. Most genes showed a low non-synonymous mutation rate (Fig. 4A), only few genes were found to have high rates of non-synonymous mutations. Compared to non-hematologic malignancies, synonymous mutation rates in our surfaceome genes were lower, possibly reflecting the more essential role of these genes in hematologic cancers (Fig. 4B).

Most notably, genes currently under investigation as immunotherapeutic targets in AML had low dN/dS-ratios, indicating their relative stability. Importantly, our putative novel targets identified in this study reliably showed a very low dN/dS-ratio, in line with genes that are of functional relevance for hematologic malignancies.

### Validation in independent patient samples and healthy donors

**Fig. 5:**
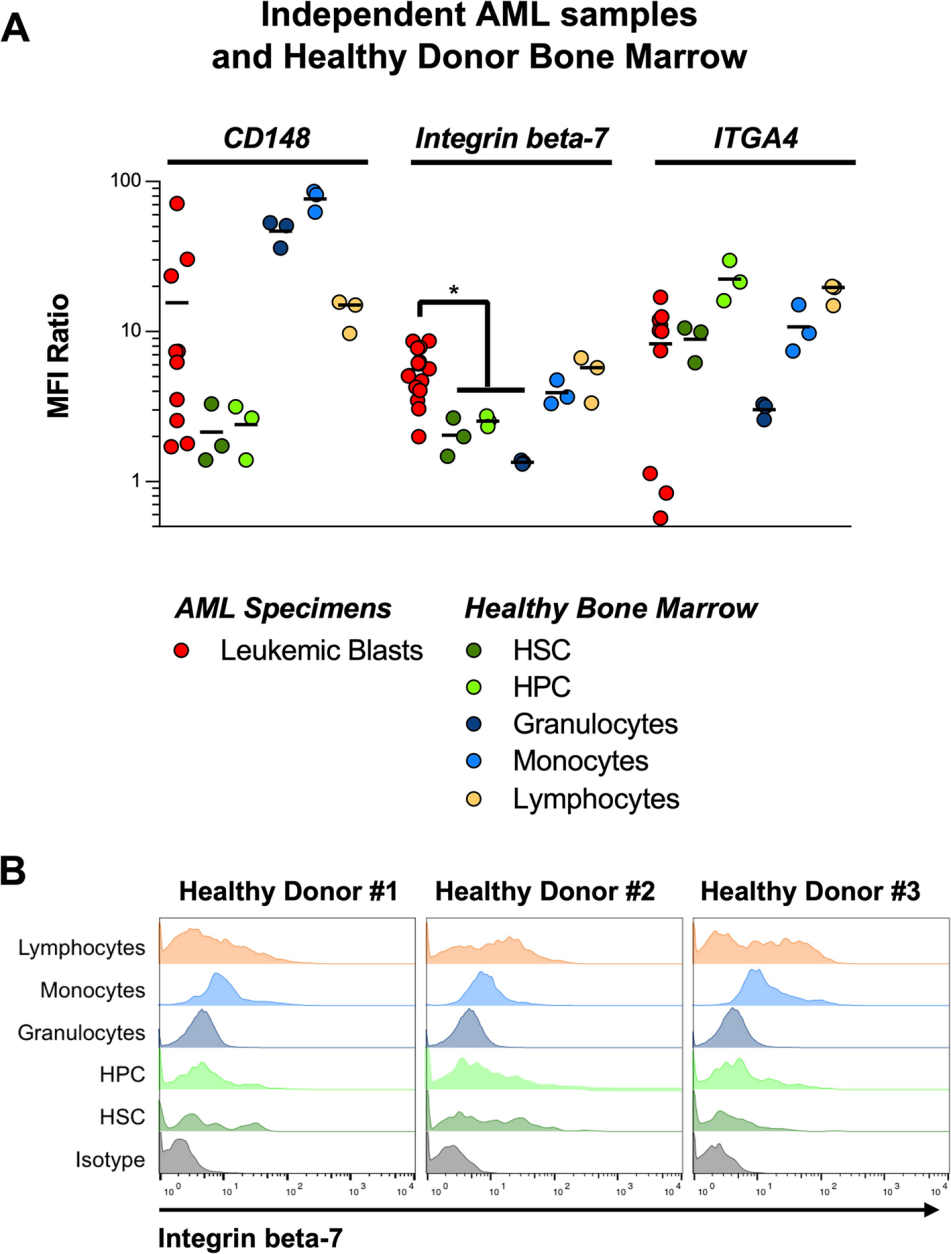
Validation of putative targets in independent samples by flow cytometry. **A** Expression of potential targets on independent patient samples and healthy donor bone marrow specimens by FACS, showing significant expression on healthy mature monocytes and granulocytes for CD148 or HSPCs for ITGA4. *p < 0.05 **B** Distribution of Integrin beta-7 on healthy donors showing favorable (low) expression on healthy hematopoietic tissues

Encouraged by these results we next validated the protein expression of a small set of novel, putative targets on a set of independent AML samples obtained during routine clinical diagnosis at our clinic. Of note, while the cell number required for our CSC-workflow required a short *in vitro* expansion, these independent patient samples used for target validation by flow cytometry were measured directly without any *in vitro* culture. We found that amongst the 5 targets investigated here, CD148, ITGA4 and to a lesser degree Integrin beta-7 (encoded by the ITGB7 gene) were consistently expressed on the cell surface in primary AML samples (Fig. 5A). Next, we measured the abundance of surface expression of these three targets on healthy hematopoietic stem and progenitor cells as well as mature granulocytes, monocytes and lymphocytes (Supplementary Figure S2). Interestingly, CD148 was found to show significant expression in both the mature lymphocyte, but more pronounced in the monocyte and granulocyte compartment, and ITGA4 showed moderate expression in the healthy HSPC compartment (Fig. 5A). These results are noteworthy, since our own selection process involved the removal of proteins found to be highly expressed based on gene expression levels in some of these subsets. This highlights the necessity to perform protein-level validation in healthy cell populations of interest even if gene expression data is available. Out of our list of putative targets, Integrin beta-7 showed the most promising combination of uniform – albeit moderate – expression in patient AML samples, and low or absent expression on healthy hematopoietic tissue (Fig. 5A and B).

## Discussion

Immunotherapeutic approaches targeting cell surface proteins have shown remarkable success in a variety of cancers. AML, has thus far remained a challenging entity for successful targeting. This is thought to be due to the choice of antigen that is being targeted, with most approaches focusing on myeloid antigens. While there has been some success in early trials targeting CD33 and CD123, on-target off-tumor toxicity has been of concern [17]. Thus, identification of novel target antigens is of great interest to the field. In this study, we optimized the cell-surface capture technology to detect the surfaceome of primary human AML. Importantly, compared to prior reports on either cell lines or xenoamplified ALL samples, our optimized system is capable of reliably detecting a high number of proteins directly from AML patient specimens, including especially less well characterized non-CD proteins. This allowed us to explore novel target antigens without the use of a-priori defined antibody panels. Using this approach, we identified 621 surface antigens expressed on our primary AML samples. We next devised a strategy to curate promising candidates for further evaluation. To serve as an ideal target antigen, putative targets have to show uniform expression, have low or absent expression on non-tumor tissue and ideally be functionally relevant to the tumor, i.e. loss of expression should confer a disadvantage to the tumor cells. We thus employed several steps to fulfill these criteria: 1) we demonstrate expression of our novel putative target antigens on a cohort of independent patient samples, 2) we demonstrate low expression levels on healthy tissue, including hematopoietic stem and progenitor cells by mRNA and protein, and 3) devise a strategy to anticipate functional relevance in tumor biology by determining the dN/dS-ratio of our putative targets.

Here, we identify three putative targets—CD148, ITGA4 and Integrin beta-7—previously not described for AML. As a membrane protein tyrosine phosphatase, CD148 is expressed on all hematopoietic lineages and plays multiple roles relevant to cell adhesion, migration, proliferation and differentiation [18–20]. Besides those functions on immune cells, CD148 has also been implicated to regulate the activation of platelets through the Src family kinases [21, 22]. It has also been considered as a potentially useful discriminating marker for the diagnosis of mantle cell lymphoma [23] to characterize mature B cell lymphoid neoplasms infiltrating blood and bone marrow [24]. Finally, CD148 has also been implicated as a prognostic marker of gastric cancer [25]. Similar to this plethora of roles, we also detected expression of CD148 on various immune cell populations including monocytes, granulocytes and lymphocytes. Therefore, we hypothesize that CD148 might be best suited as a therapeutic target in combinatorial strategies [26].

ITGA4 (CD49d) acts as a part of the very late antigen-4 (VLA-4) and has been studied in the context of chronic lymphocytic leukemia (CLL), where it has been found to represent an adverse prognostic marker [27–29]. Similarly, ITGA4 has been shown to correlate with prognosis in patients with early Oral Squamous Cell Carcinoma [30]. In our analysis, expression of ITGA4 was less homogenous, similarly to the reported binary expression pattern in CLL. However, ITGA4 expression was also detected on several healthy hematopoietic cells, which might impact the suitability of ITGA4 as a single target antigen. Studies on the prognostic role of ITGA4 expression in AML are very limited [31], thus we expect future studies to illuminate its role further.

Integrin beta-7 has been suggested to play a role in high-risk multiple myeloma and overexpression of Integrin beta-7 has been associated with DNA-hypomethylation [32]. In addition to its cell adhesion, migration and homing roles, Integrin beta-7 has also been suggested to participate in regulating the immune microenvironment as well as suppressing cell proliferation through the glycolysis/glucose metabolism pathway [33, 34]. However, the exact role of Integrin beta-7 specifically in AML is unknown. Interestingly, proteins encoded by ITGB7 and ITGA4 form a heterodimer known as LPAM-1, thus likely explaining the coincidence on our list of putative targets. At the same time, Integrin beta-7 is an exclusive dimerization partner ITAE [35]. Therefore, targeting of heterodimers formed by Integrin beta-7 could improve specificity of immunotherapeutic approaches.

We aimed to determine the feasibility of our approach in a diverse set of primary patient samples, representing a variety of genetic backgrounds and wanted to explore whether we might be able to identify common target antigens using this approach. Further studies will have to determine if genetically defined subgroups of AML cases will reveal differentially expressed target antigens. Our method represents a proof-of-concept approach to evaluate this in future studies.

Taken together, this study established an improved proteomics assay for the efficient interrogation of the entire surface proteome (surfaceome) of primary acute myeloid leukemia without the necessity of xenograft amplification. We demonstrate how these datasets can be integrated with other modalities to comprehensively explore putative novel antigens. In our dataset, Integrin beta-7 showed the most promising constellation of homogenous expression in our validation cohort and low expression on healthy tissues. Thus, Integrin beta-7 is a potential antigen of interest for AML that has not been proposed yet, warranting further study.

## Conclusion

We developed an integrated approach which allows for unbiased, genome-wide screening of all three modalities – mutation, transcription and surface expression – for target discovery in immunotherapy of AML directly from patient specimens. Using this approach, we identify Integrin beta-7 as a potential target antigen for the immunotherapy of AML and validate this candidate antigen in primary AML patient specimens.

## Supplementary Information

**Additional file 1: Table S1.** Clinical characteristics of patients included in study.

**Additional file 2: Table S2.** Shortlist of 76 putative targets.

**Additional file 3: Table S3.** AML Surfaceome and gene expression values from healthy tissues.

**Additional file 4: Table S4.** Antibody panel for flow cytometric validation.

**Additional file 5: Figure S1.** Expression patterns and cut-off determination for AML Surfaceome. A: Expression of Surfaceome genes in hematopoietic stem cells in the bone marrow (HSCs – top panel), early hematopoietic progenitors in the bone marrow (HPC, middle panel) and CD14+ monocytes (bottom panel). Data derived from BloodSpot database (HemaExplorer). Cut-off used for filtering of Surfaceome candidates shown (calculated as the mean + SD). B: Expression of Surfaceome genes in non-hematopoietic tissue, displaying average non-heme tissue expression (top panel) or maximal non-heme tissue expression (from Genotype-Tissue Expression Project - GTEx). Cut-off used for filtering of Surfaceome candidates shown (calculated as the mean + SD).

**Additional file 6: Figure S2.** Gating of subpopulations for FACS validation of putative Surfaceome targets. A: Expression of putative Surfaceome markers was assessed on healthy bone marrow specimens gating for both mature (lymphocyte, monocyte, granulocyte) as well as progenitor (hematopoietic stem cell “HSC”, hematopoietic progenitor cell “HPC”) populations. B: Gating of “Blast” population in leukemia specimens. ”Blasts” were gated based on dim/intermediate CD45 expression.

**Additional file 7: Figure S3. **Number of proteins identified in technical replicates. Venn Diagram representing number of unique proteins identified by our modified CSC workflow from a xeno-amplified AML specimen (PDX1) processed separately. >90% of proteins were identified in both runs, suggesting highly robust identification of the surface proteome.

## Data Availability

All primary data generated in this study is supplied as supplementary information.
