# Supplementary material for: Integrated multiomic approach for identification of novel immunotherapeutic targets in AML
Source: Biomark Res. 2022 Jun 10;10:43. doi: 10.1186/s40364-022-00390-4 (PMC9185890; doi:10.1186/s40364-022-00390-4)
Supplement: Supplementary file 1 — Additional file 1: Table S1. Clinical characteristics of patients included in study. [file 40364_2022_390_MOESM1_ESM.docx]

**Supplementary Table S1: Clinical Characteristics of Patients Included in Study**

| Sample | Sex | Age (Y) | FAB | Disease Stage | Cytogenetics | NPM1 | FLT3-ITD | ELN | BM/PB |
| --- | --- | --- | --- | --- | --- | --- | --- | --- | --- |
| PDX 1 | F | 47 | M4 | Relapse  after SCT | 46,XX, ins(10;11)  (p12;q23q23) | WT | WT | Adv | BM |
| PDX 2 | M | 57 | M4 | Primary Diagnosis | 46,XY,t(6;11)  (q27;q23) | WT | WT | Adv | BM |
| PP 01 | F | 35 | M1 | Primary Diagnosis | 46,XX | WT | WT | Fav | BM |
| PP 03 | F | 28 | M5 | Primary Diagnosis | 46,XX,t(11;19)  (q23;p13.3) | WT | WT | Adv | BM |
| PP 04 | M | 55 | NA | Relapse | 46,XY | WT | WT | NA | PB |
| PP 05 | F | 53 | NA | Primary Diagnosis | 46,XX | Mut | ITD | NA | BM |
| PP 06 | F | 31 | NA | Primary Diagnosis | 46,XX,der(8)(8;21)(q22;q22),der(22)t(8;22)(q22;q1?3)[21] | WT | WT | NA | BM |

PDX (patient-derived xenograft); PP (Primary Patient); F (female); M (male); FAB (French–American–British classification system); SCT (stem cell transplantation); NPM1 (nucleophosmin-1); WT (wildtype); Adv (adverse); Mut (mutated); FLT3 (Fms-related tyrosine kinase 3); ITD (internal tandem duplication); ELN (European LeukemiaNet classification system); PB (peripheral blood); BM (bone marrow); NA (not available)
