# Supplementary material for: Integrated multiomic approach for identification of novel immunotherapeutic targets in AML
Source: Biomark Res. 2022 Jun 10;10:43. doi: 10.1186/s40364-022-00390-4 (PMC9185890; doi:10.1186/s40364-022-00390-4)
Supplement: Supplementary file 5 — Additional file 5: Figure S1. Expression patterns and cut-off determination for AML Surfaceome. A: Expression of Surfaceome genes in hematopoietic stem cells in the bone marrow (HSCs – top panel), early hematopoietic progenitors in the bone marrow (HPC, middle panel) and CD14+ monocytes (bottom panel). Data derived from BloodSpot database (HemaExplorer). Cut-off used for filtering of Surfaceome candidates shown (calculated as the mean + SD). B: Expression of Surfaceome genes in non-hematopoietic tissue, displaying average non-heme tissue expression (top panel) or maximal non-heme tissue expression (from Genotype-Tissue Expression Project - GTEx). Cut-off used for filtering of Surfaceome candidates shown (calculated as the mean + SD). [file 40364_2022_390_MOESM5_ESM.pdf]

A

	HSC_BM	early HPC_BM	CD14+ monocytes
Mean	6.414	6.461	7.006
SD	2.651	2.581	3.137

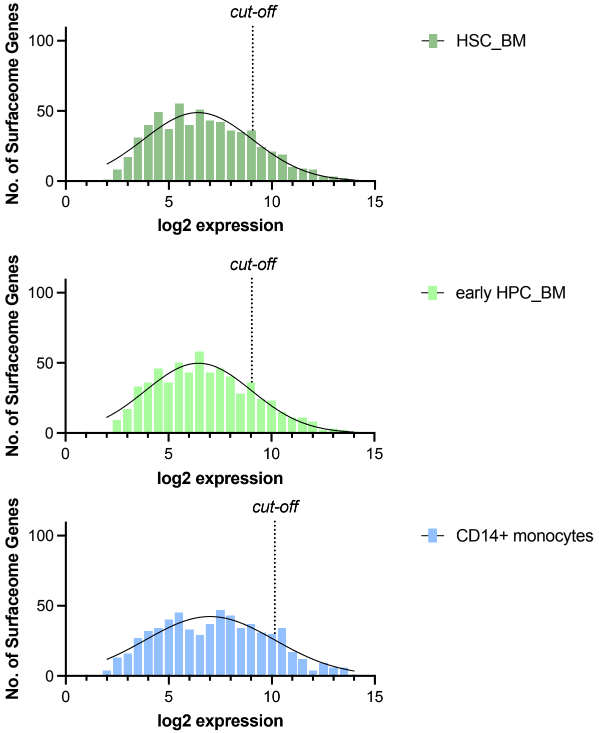

B

	Average Non-Heme Tissue Expression	Maximal Non-Heme Tissue Expression
Mean	2.803	4.586
SD	2.368	2.008

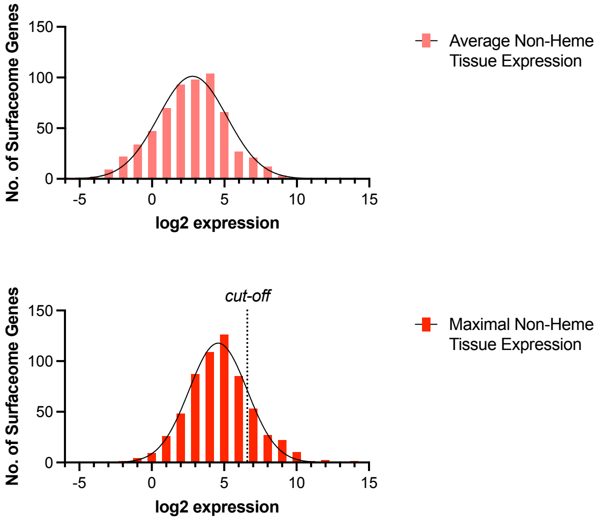

Supplementary Figure S1. Expression patterns and cut-off determination for AML Surfaceome. A: Expression of Surfaceome genes in hematopoietic stem cells in the bone marrow (HSCs – top panel), early hematopoietic progenitors in the bone marrow (HPC, middle panel) and CD14+ monocytes (bottom panel). Data derived from BloodSpot database (HemaExplorer). Cut-off used for filtering of Surfaceome candidates shown (calculated as the mean + SD). B: Expression of Surfaceome genes in non-hematopoietic tissue, displaying average non-heme tissue expression (top panel) or maximal non-heme tissue expression (from Genotype-Tissue Expression Project - GTEx). Cut-off used for filtering of Surfaceome candidates shown (calculated as the mean + SD).
