# Supplementary material for: Integrated multiomic approach for identification of novel immunotherapeutic targets in AML
Source: Biomark Res. 2022 Jun 10;10:43. doi: 10.1186/s40364-022-00390-4 (PMC9185890; doi:10.1186/s40364-022-00390-4)
Supplement: Supplementary file 6 — Additional file 6: Figure S2. Gating of subpopulations for FACS validation of putative Surfaceome targets. A: Expression of putative Surfaceome markers was assessed on healthy bone marrow specimens gating for both mature (lymphocyte, monocyte, granulocyte) as well as progenitor (hematopoietic stem cell “HSC”, hematopoietic progenitor cell “HPC”) populations. B: Gating of “Blast” population in leukemia specimens. ”Blasts” were gated based on dim/intermediate CD45 expression. [file 40364_2022_390_MOESM6_ESM.pdf]

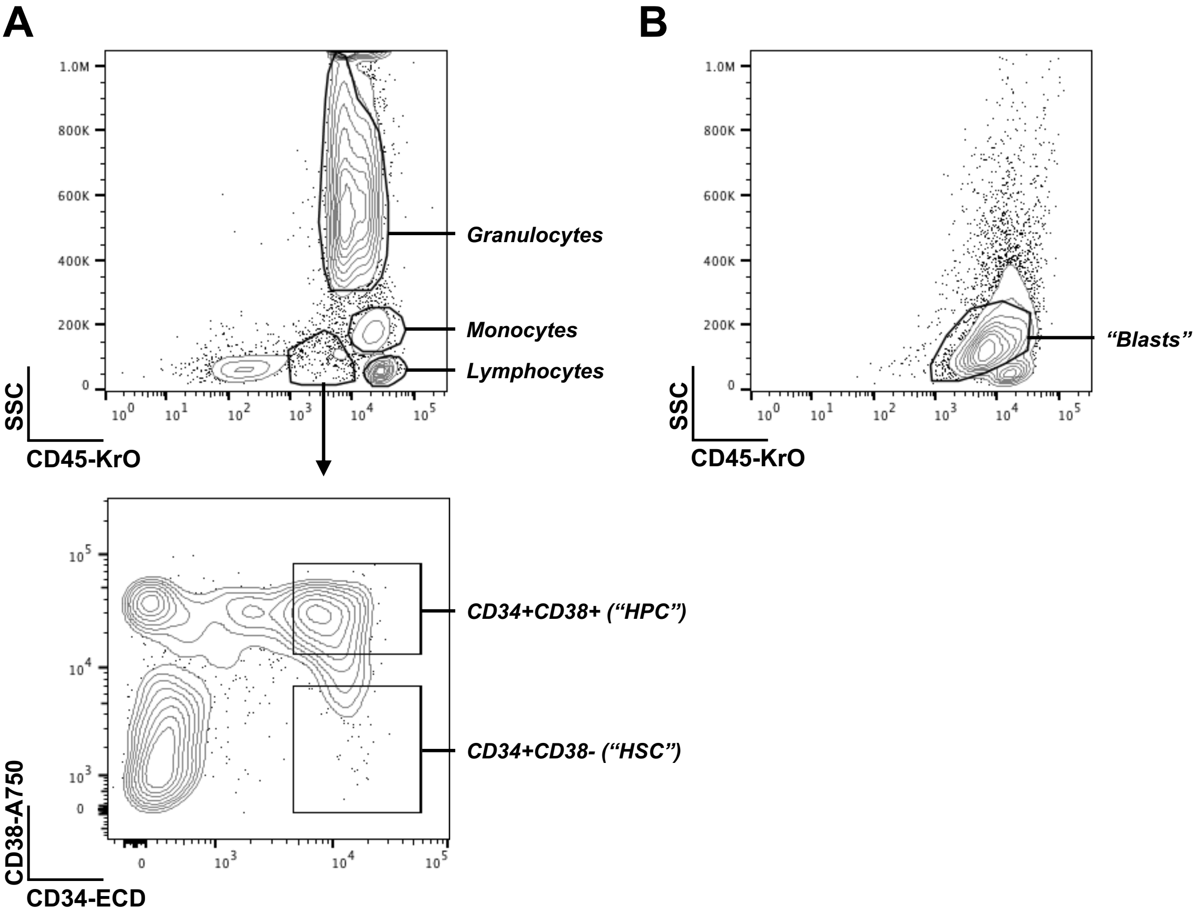

Supplementary Figure S2. Gating of subpopulations for FACS validation of putative Surfaceome targets. A: Expression of putative Surfaceome markers was assessed on healthy bone marrow specimens gating for both mature (lymphocyte, monocyte, granulocyte) as well as progenitor (hematopoietic stem cell “HSC”, hematopoietic progenitor cell “HPC”) populations. B: Gating of “Blast” population in leukemia specimens. ”Blasts” were gated based on dim/intermediate CD45 expression.
