# Supplementary material for: Integrated multiomic approach for identification of novel immunotherapeutic targets in AML
Source: Biomark Res. 2022 Jun 10;10:43. doi: 10.1186/s40364-022-00390-4 (PMC9185890; doi:10.1186/s40364-022-00390-4)
Supplement: Supplementary file 7 — Additional file 7: Figure S3. Number of proteins identified in technical replicates. Venn Diagram representing number of unique proteins identified by our modified CSC workflow from a xeno-amplified AML specimen (PDX1) processed separately. >90% of proteins were identified in both runs, suggesting highly robust identification of the surface proteome. [file 40364_2022_390_MOESM7_ESM.pdf]

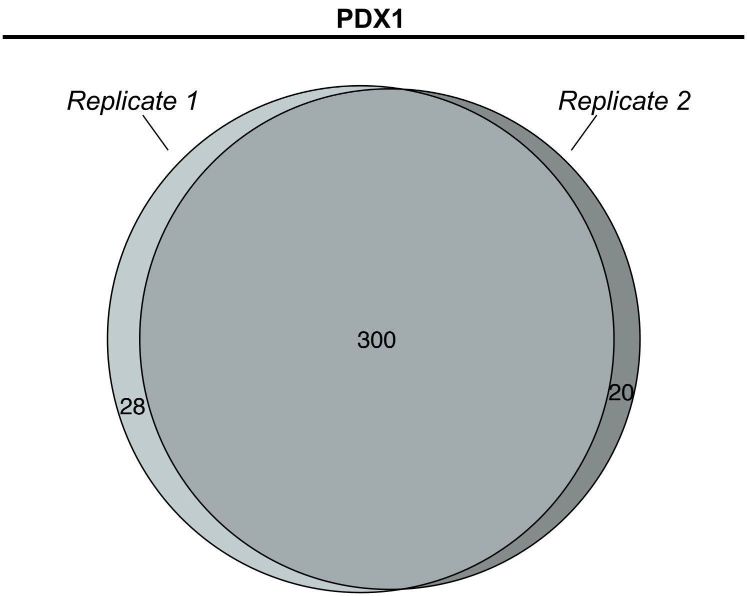

Supplementary Figure S3. Number of proteins identified in technical replicates. Venn Diagram representing number of unique proteins identified by our modified CSC workflow from a xeno-amplified AML specimen (PDX1) processed separately. >90% of proteins were identified in both runs, suggesting highly robust identification of the surface proteome.
